# Parathormone Levels in a Middle-Eastern Healthy Population Using 2^nd^ and 3^rd^ Generation PTH Assays

**DOI:** 10.1155/2020/6302861

**Published:** 2020-02-21

**Authors:** Marie-Hélène Gannagé-Yared, Marie-Noëlle Kallas-Chémaly, Ghassan Sleilaty

**Affiliations:** ^1^Endocrinology Department, Faculty of Medicine, Saint Joseph University, Beirut, Lebanon; ^2^Department of Biostatistics, Faculty of Medicine, Saint-Joseph University, Beirut, Lebanon; ^3^Clinical Research Center, Faculty of Medicine, Saint-Joseph University, Beirut, Lebanon

## Abstract

**Background:**

The purpose of the current study is to determine PTH reference values in vitamin-D-replete Lebanese adults using 2^nd^ and 3^rd^ generation PTH assays and to look at the factors that affect PTH variations.

**Methods:**

Fasting PTH was measured using 2^nd^ and 3^rd^ generation Diasorin PTH assays in 339 vitamin-D-replete healthy subjects aged 18 to 63 years (230 men and 109 women) who have normal calcium levels and an eGFR ≥60 ml/mn. 25-OH vitamin D (25(OH)D) was measured using the Diasorin assay.

**Results:**

For the 2^nd^ PTH generation, median (IQR) levels were 48.9 (34.9–66.0) pg/ml, and its 2.5^th^–97.5^th^ percentile values were 19.7–110.5 pg/ml for 25(OH)D values between 20 and 30 ng/ml, and 19.7–110.7 pg/ml for 25(OH)D values ≥30 ng/ml. For the 3^rd^ PTH generation, the median (IQR) values were 23.9 (17.7–30.5) pg/ml, and its 2.5^th^–97.5^th^ percentile values were, respectively, 9.2 and 50.2 pg/ml for 25(OH)D values between 20 and 30 ng/ml, and 8.4 and 45.4 pg/ml for 25(OH)D values ≥30 ng/ml. The median (IQR) serum 25(OH)D levels were 27.5 (23.8–32.7) ng/ml. 2^nd^ and 3^rd^ generation PTH values are strongly correlated (*r* = 0.96, *p* < 0.0001), but poorly concordant (Lin's concordance coefficient 0.365, 95% CI: 0.328–0.401) with observations beyond the 95% Bland–Altman limits of agreement. 2^nd^ and 3^rd^ generation PTH levels did not differ according to gender and were significantly correlated with age but not with 25(OH)D and serum calcium levels.

**Conclusion:**

Lebanese adult healthy subjects have higher 2^nd^ and 3^rd^ generation PTH levels compared with the reference range provided by the manufacturer. The reference range was not influenced by changing the 25(OH)D cutoff. The clinical significance of the higher PTH levels in our population should be investigated.

## 1. Introduction

In clinical practice, assessing parathormone (PTH) concentration is important in exploring calcium/phosphorus metabolism disorders and in monitoring patients suffering from chronic kidney disease. Unfortunately, this task is complex despite the advent of automated laboratory assays. In fact, there are considerable variations in PTH values obtained from different assays, even when provided by the same manufacturer [1, 2]. This variability is mainly related to the assay measurement of different PTH fragments. Older PTH assays, called second (2^nd^) generation assays or “intact” PTH, are known to measure not only the 84 amino acid molecules but also to cross-react with a truncated PTH fragment called the 7–84 PTH, whereas the more recently introduced assays, named the third (3^rd^) generation assays, do not measure this truncated fragment, but may cross-react with another fragment called amino-PTH [3]. This difference explains the higher PTH values obtained with 2^nd^ generation assays compared with the 3^rd^ generation ones in healthy subjects [4, 5] as well as in subjects with chronic renal failure [4, 6, 7].

Establishing a normal reference range for PTH is usually based on the values measured in 95% of healthy individuals after ruling out potential confounding factors since the PTH level is affected by multiple factors, such as vitamin D status, age, and renal function [1, 8–10]. Therefore, vitamin D deficiency as well as renal failure, both conditions leading to secondary hyperparathyroidism, should be ruled out or taken into consideration when establishing a reference range of PTH [11]. Other interfering factors such as calcium/phosphorus disorders should be also excluded [12]. Reference values for PTH are available in European [13, 14], United States [15], and Chinese populations [16]. However, normative PTH values are lacking in the Middle-East, more particularly in Lebanon, a part of the world known for its high prevalence of vitamin D deficiency [17, 18]. In addition, reference values were mainly established using either 2^nd^ or 3^rd^ generation PTH assays. Few studies compared both assays in healthy adults [4, 5, 19] and included only a small number of subjects [4, 19]. It is thus unclear if there is a difference in normative values between both assays. Moreover, the concordance between both assays has only been studied in one study with a good concordance in healthy patients [4].

The purpose of the current study is first to determine PTH reference values in Lebanese adults who are vitamin-D-replete and have a normal renal function using both 2^nd^ and 3^rd^ generation PTH assays; the second is to identify factors that may affect PTH variations.

## 2. Materials and Methods

### 2.1. Patients

The study included a total of 339 subjects (230 women and 109 men) aged 18 to 63 years, who presented at the Laboratory of Hôtel-Dieu de France Hospital (one of the biggest university hospitals in the Beirut area) for a routine biochemistry evaluation including calcium, creatinine, and 25(hydroxyvitamin) D (25(OH)D) measurements. Inclusion criteria to measure PTH in these subjects were normal serum calcium (values between 2.10 and 2.56 mmol/L), estimated glomerular filtration rate (eGFR) ≥60 mL/min/1.73 m^2^, and 25(OH)D levels ≥20 ng/ml. The 20 ng/ml cutoff value supported by the Institute of Medicine (IOM) report was used to define optimal vitamin D status [20].

### 2.2. Biochemical Measurements

Morning fasting blood specimens were collected in dry tubes, then centrifuged within 30 min after venipuncture, and analyzed the day of sampling for calcium, creatinine, and 25(OH)D measurements. Part of the serum was immediately frozen and stored at −20°C for later PTH measurements. Only samples with a 25(OH)D ≥20 ng/ml were subsequently analyzed within one month for both 2^nd^ and 3^rd^ PTH measurements. Measurements of total calcium and creatinine levels were performed using a Vitros 5.1 FS automate (Ortho-Clinical Diagnostics, Inc. Raritan, New Jersey). The eGFR was assessed using the Chronic Kidney Disease Epidemiology Collaboration (CKD-EPI) equation [21].

### 2.3. 25(OH)D and PTH Assays

PTH and 25(OH)D measurements were done in batches on the LIAISON XL (DiaSorin, Stillwater, MN, USA). Serum 25(OH)D was measured using a direct competitive chemiluminescence immunoassay (CLIA). The observed reference range is 9.3–47.9 ng/ml. The lowest reported value is 4 ng/mL, and the interassay coefficient of variation (CV) <20%. The DiaSorin Liaison PTH 2^nd^ and 3^rd^ generation assays were used to measure PTH values. The PTH 2^nd^ generation assay is a modified 2-step, 2-site sandwich assay using 2 polyclonal antibodies to detect intact PTH, and the expected range provided by the kit comprised between 14.5 and 87.1 pg/ml corresponding to the 2.5^th^ and the 97.5^th^ percentiles. The DiaSorin Liaison PTH 3^rd^ generation or 1–84 PTH assay is also a 2-step, 2-site sandwich assay that uses a first antibody that is highly specific for the N terminus of 1–84 PTH to ensure no cross reactivity with fragments such as 7–84 PTH, while the second polyclonal antibody is targeted against the C-terminal region of the 1–84 molecule. The expected reference range provided by the kit comprises between 6.5 and 36.8 pg/ml. Interassay CV was less than 15% for both PTH assays.

### 2.4. Statistical Analysis

The distribution of 2^nd^ and 3^rd^ generation PTH values was checked using Kolmogorov–Smirnov (KS) and Shapiro–Wilk (SW) tests, with additional visual inspection of quartile-quartile (Q-Q) plots. Logarithmic transformation (natural logarithm) was applied to 2^nd^ and 3^rd^ generation PTH values (labeled Ln (PTH 2G) and Ln (PTH 3G), respectively).

The native variables with skewed distribution were expressed as median with its interquartile range (quartile 1–quartile 3) (Table 1) and percentiles 2.5% and 97.5% (Table 2). For the transformed variables satisfying normality assumptions, the mean and the standard deviation were calculated.

Correlation between Ln (PTH 2G) and Ln (PTH 3G) was estimated using Pearson correlation coefficient, and its 95% confidence interval was calculated by bootstrapping performed on 1000 samples. *R*^2^ was also calculated.

Agreement between Ln (PTH 2G) and Ln (PTH 3G) was studied using Bland–Altman method, and Lin's concordance correlation coefficient was calculated using a macro developed by M. Garcia-Granero (http://gjyp.nl/marta/Lin.sps), providing an asymptotic 95% confidence interval.

The distributions of 2^nd^ and 3^rd^ generation PTH values were compared for 25(OH)D values between 20 and 30 ng/ml and values ≥30 ng/ml using Mann–Whitney *U* test. Percentiles 2.5% and 97.5% of PTH 2G and PTH 3G of the current study were further compared with expected values provided with the kits by the manufacturers, respectively, using 95% bias corrected accelerated (BCa) confidence intervals built by bootstrapping on 1000 samples. Correlation between 2^nd^ and 3^rd^ generation PTH values and 25(OH)D, serum calcium levels, and CKD EPI eGFR values relied on Spearman's correlation coefficient and its BCa 95% confidence interval. Kappa statistic for agreement between nominal variables was also calculated.

The statistical analysis was performed using IBM SPSS (IBM Corp.; SPSS Statistics for Windows v22.0, Armonk, NY, USA).

### 2.5. Ethical Issues

The study was approved by the Ethics Committee of our university hospital (CEHDF 1247). The authors have complied with the World Medical Association Declaration of Helsinki regarding ethical conduct of research involving human subjects.

## 3. Results

The baseline characteristics of the sample are shown in Table 1. Female subjects were slightly younger than males (42.5 ± 11.5 vs. 45.7 ± 12.2 years, *p*=0.021), with a lower serum creatinine (58.6 ± 9.0 *μ*mol/L vs. 76.2 ± 12.6 *μ*mol/L, *p* < 0.001) and marginally higher eGFR (106.7 ± 13.4 vs. 102.5 ± 13.9 mL/min/1.73 m^2^, *p*=0.008) that disappeared after adjusting for age (*p*=0.135). In the overall sample, the mean serum calcium level was 2.40 ± 0.08 mmol/L.

### 3.1. 25(OH)D and PTH Measurements and Reference Range of Serum PTH Concentrations

The median serum 25(OH)D value was 27.5 (23.8–32.7) ng/ml with no significant difference according to gender (*p*=0.840). 64.6% of the subjects had 25(OH)D values between 20 and 30 ng/ml, and 35.6% had 25(OH)D values ≥30 ng/ml.

**Figure 1:**
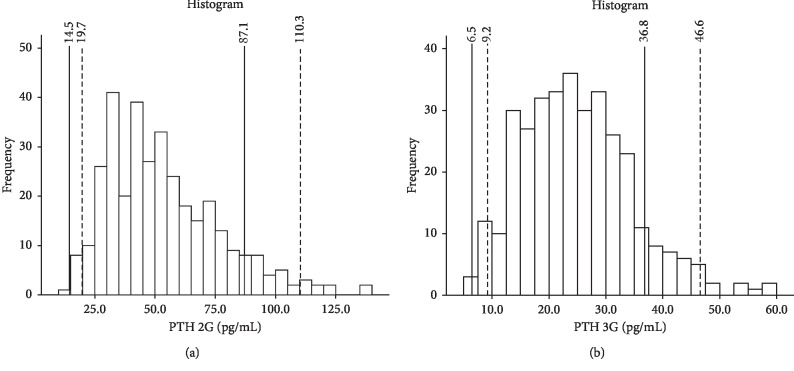
Histograms showing the distribution of 2^nd^ generation PTH values (a) and 3^rd^ generation PTH values (b). Uninterrupted vertical lines represent percentiles 2.5% and 97.5% as provided by the manufacturer. Dashed vertical lines represent actual percentiles 2.5% and 97.5% from the current series.

**Figure 2:**
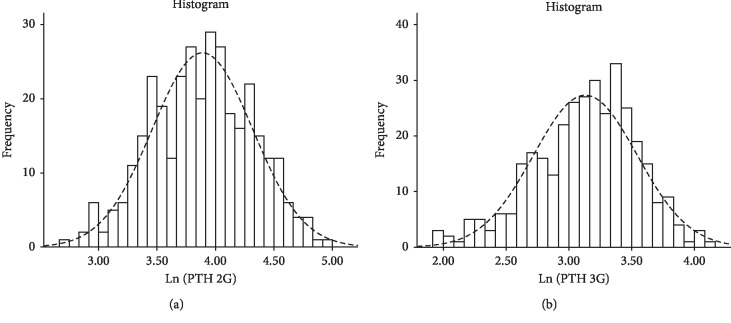
Histograms showing the distribution of log-transformed values of 2^nd^ generation PTH values (a) and 3^rd^ generation PTH values (b). Dashed lines represent a superimposed normal distribution.

2^nd^ and 3^rd^ generation PTH showed a significant departure from normality and a right-skewed distributions (Figure 1). Therefore, their natural logarithm transforms (Ln (PTH 2G) and Ln (PTH 3G)), which satisfied normality assumptions, were used in subsequent analysis (Figure 2).

The medians of 2^nd^ and 3^rd^ generation PTH assays were 48.9 (34.9–66.0) pg/ml and 23.9 (17.7–30.5) pg/ml, respectively (Table 1).

The percentiles 2.5% and 97.5% for 2^nd^ and 3^rd^ generation PTH were (19.7–110.5) pg/ml and (9.2–46.6) pg/ml, respectively (Table 2). The distributions of 2^nd^ and 3^rd^ generation PTH according to dichotomized 25(OH)D values (between 20 and 30 ng/ml and ≥30 ng/ml) showed no statistical difference between both groups (*p*=0.696 and *p*=0.917, respectively) (Table 2). When compared to the reference 97.5% percentile values provided by the manufacturer, respectively, 10.0% and 11.2% of the subjects had their 2^nd^ and 3^rd^ generation PTH values beyond the reference percentile (respectively, 95% CI: 7.4%–13.0% and 8.1%–14.4%), both comparable but significantly different from the threshold specified by the manufacturer. Out of the 34 subjects with 2^nd^ generation PTH above the reference 97.5% percentile, 4 (11.8%) had normal 3^rd^ generation PTH values. Conversely, out of the 38 subjects with 3^rd^ generation PTH above the reference 97.5% percentile, 8 (21.1%) had normal 2^nd^ generation PTH values (Kappa measure of agreement = 0.814 ± 0.052), corroborating the Bland–Altman method findings. None of the subjects had both 2^nd^ and 3^rd^ generation PTH values below the 2.5% percentile reference values.

**Figure 3:**
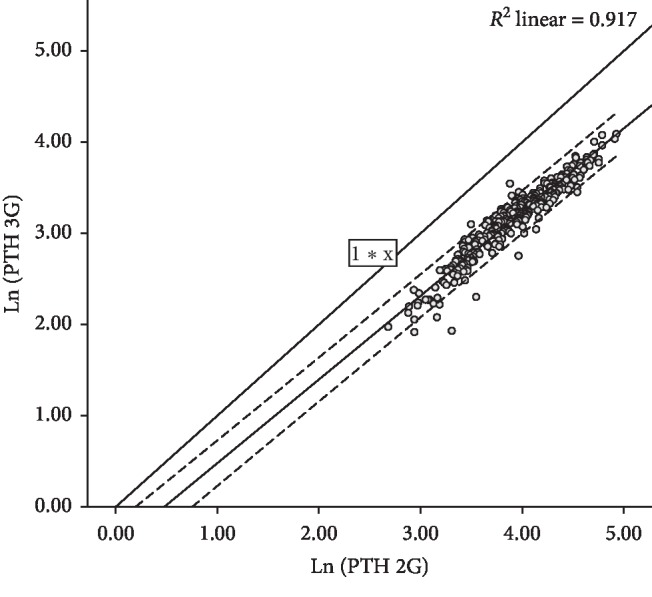
Scatterplot showing the correlation between log(2^nd^ generation PTH) (Ln (PTH 2G)) values and log(3^rd^ generation PTH) (Ln (PTH 3G)) values. The dashed lines represent 95% confidence limits for correlation. The (1 *∗* *x*) line represents the line of perfect concordance (that is, *y* = *x*, where Lin's coefficient equals 1).

**Figure 4:**
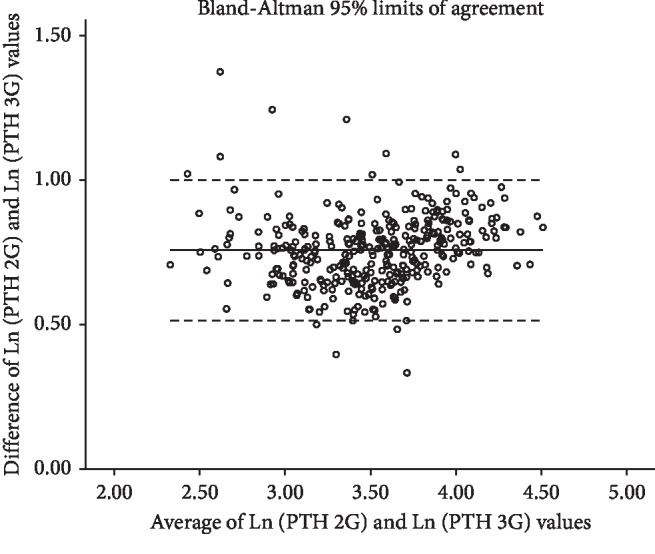
Bland–Altman plot showing the agreement between log(2^nd^ generation PTH) (Ln (PTH 2G)) values and log(3^rd^ generation PTH) (Ln (PTH 3G)) values. Central horizontal line represents the mean bias. The dashed horizontal lines represent 95% limits of agreement.

While the Pearson correlation coefficient between Ln (PTH 2G) and Ln (PTH 3G) was high, reaching 0.957 (95% CI: 0.945–0.968, *p* < 0.0001) as shown in Figure 3, the agreement between Ln (PTH 2G) and Ln (PTH 3G) was low since Lin's concordance correlation coefficient was only 0.365 (95% CI: 0.328–0.401). The Bland–Altman graphical analysis is consistent with these findings since a significant number of pairs of observations are beyond 95% limits of agreement (Figure 4), with a mean bias of 0.758 on the logarithmic scale (2.13 pg/ml on natural scale).

### 3.2. Relationship of Serum PTH Level with Gender and Age

No significant differences in 2^nd^ and 3^rd^ generation PTH levels were observed according to gender (*p*=0.788 and *p*=0.798, respectively, Table 1). There was a significant correlation between 2^nd^ generation PTH levels and age (Pearson correlation coefficient for age and Ln (PTH 2G) = 0.270 (95% CI: 0.170–0.370, *p* < 0.001)), as well as between 3^rd^ generation PTH levels and age (Pearson correlation coefficient for age and Ln (PTH 3G) = 0.267 (95% CI: 0.161–0.373, *p* < 0.001)).

### 3.3. Relationship of Serum PTH Level with 25(OH)D and Other Biological Parameters

There was no significant correlation between 2^nd^ generation PTH levels and 25(OH)D (Spearman's correlation coefficient = −0.030, 95% CI: −0.137–0.073, *p*=0.584) nor between 3^rd^ generation PTH levels and 25(OH)D (Spearman's correlation coefficient −0.002, 95% CI: −0.108–0.094, *p*=0.968).

Likewise, there was no significant correlation between 2^nd^ generation PTH levels and serum calcium levels (Spearman's correlation coefficient = −0.087, 95% CI: −0.192–0.024, *p*=0.109), nor between 3^rd^ generation PTH levels and serum calcium levels (Spearman's correlation coefficient = −0.094, 95% CI: −0.199–0.019, *p*=0.085). There was a weak but statistically significant inverse correlation between CKD-EPI eGFR levels and both 2^nd^ and 3^rd^ generation PTH levels (Spearman's correlation coefficients were −0.107, 95% CI: −0.207–0.001, *p*=0.049 and −0.111, 95% CI: −0.205–0.002, *p*=0.042, respectively).

## 4. Discussion

In this study, we established the reference ranges for PTH in 339 Lebanese subjects using the Diasorin 2^nd^ and 3^rd^ generation PTH assays. These reference ranges were established in vitamin-D-replete subjects (25(OH)D ≥20 ng/ml) after excluding subjects with abnormal calcium levels and low eGFR. We found that the 97.5% percentiles for 2^nd^ and 3^rd^ generation PTH are, respectively, 110.5 and 46.6 pg/ml, that is, 26.8% higher than the upper limit of normal (ULN) reference values of 87.1 and 36.8 pg/ml provided by the manufacturer.

Different authors evaluated the reference values for PTH in vitamin-D-replete subjects. As an example, using the Cobas/Elecsys Roche 2^nd^ generation PTH kit, Cavalier et al. [22] found in 240 healthy Belgian subjects a ULN PTH value of 50 pg/ml which is lower than the 65 pg/ml value provided by the manufacturer. Similarly, using the same assay, Touvier et al. [10] found that the plasma PTH ULN is of 45.3 pg/ml in 1824 French adults recruited from the SUVIMAX study. In contrast, two other studies found higher ULN reference values with the same assay. The first one [8] was performed on a Danish population composed of 2316 women and reported a ULN of 67 pg/ml, while the second one [16] was performed on 1436 healthy Chinese subjects, and the ULN reference value was 70 pg/ml. Moreover, using the 3^rd^ PTH Diasorin assay, Souberbielle [13] found in 898 healthy French adults that the ULN value was 28.9 pg/ml, a value that is also lower than the value provided by the manufacturer. Finally, in a United States (US) study [15], the authors compared the performance characteristics of six 2^nd^ PTH assays and found that the ULN values of their population are comparable or higher than the one established by the manufacturers. The differences between the French and Belgian studies [10, 13, 22] on one side and the other studies on the other are unclear. Since age is a significant determinant of PTH, that difference could be explained by the wider age range of the Danish study [8] (which includes women aged up to 84) compared with the French studies [10, 13] in which none [10] or very few [13] of the included subjects were older than 65. Other factors such as a later daytime and a nonfasting blood collection could also contribute to the higher PTH levels in the Danish study. Finally, ethnic differences could explain the higher PTH values observed in the Chinese study [16] as well as in the present study. Consequently, PTH ULN reference values seems to differ between different countries, with the lowest reference values being observed in Belgium and France. In our opinion, the higher ULN reference values observed in our sample as compared with western countries might be related to the higher prevalence of vitamin D deficiency [17, 18]. In fact, it is possible that in some patients, a persistent vitamin D deficiency and long-lasting secondary hyperparathyroidism induces an autonomous secretion of PTH (i.e., tertiary hyperparathyroidism) leading to parathyroid adenoma or hyperplasia [23]. The mechanism behind this finding could be, as suggested by Souberbielle [23], the desensitization of parathyroid vitamin D receptor leading to a decreased expression of the calcium sensor receptor and therefore to a shift in the calcium set-point. The reason why some but not others may develop this secondary parathyroid growth is unclear.

When comparing the 2 different PTH generation assays in our sample, a strong positive correlation was noted, but the agreement between them was low according to Lin's concordance correlation coefficient, thus suggesting that the assays are not interchangeable. Few studies compared the two PTH generations in the same sample: in French healthy subjects [4] PTH concentration did not differ according to the generation of the used kits, except for an overestimation with the 2^nd^ generation assays for values beyond 200 pg/ml. In another study [5], there was poor concordance between the methods; moreover, this worsened in the higher range of measurements.

Because PTH may be increased in patients with vitamin D deficiency and decreased in vitamin-D-replete subjects, exclusion of patients with vitamin D insufficiency from the reference population for PTH values is mandatory. Since the 25(OH)D cutoff that should be used (20 versus 30 ng/ml) is not clearly established, we compared the reference values based on both 25(OH)D cutoffs. We found that the PTH ULN did not differ no matter they used cutoff, corroborating the US study by La'ulu [15] in which the Diasorin assay was also used. The fact that no significant inverse relationship was found between 25(OH)D and PTH might be due to a vitamin-D-replete status in our population and suggests that a cutoff of 20 ng/ml might be enough to decrease PTH levels until it is stabilized. This does not exclude the possibility that higher 25(OH)D levels are requested to ensure this decrease.

We finally searched for a possible relationship between PTH and both age and gender. Similarly to other studies [8], we found a positive relationship between age and PTH without any difference according to gender. Other studies found an increase in PTH values with age in both Turkish [24] and US populations [25]. In addition, in Turkish females, PTH values were higher than those in males [24] while other studies, similarly to ours, did not show this gender difference [10].

The reason why PTH is higher in our population compared to western population is unclear. As explained above, and since the Lebanese population is at a high risk for vitamin D deficiency [17, 18], prolonged secondary hyperparathyroidism may lead to higher PTH values in our population even after vitamin D repletion. As a result, adopting different reference range values in our population may modify our therapeutic decisions. The obvious consequence of this finding is that, in clinical practice, much less patients will be detected as having normocalcemic primary hyperparathyroidism (PHPTH). Another possibility could be the presence of patients with normocalcemic PHPTH amongst our study population. Here, the only way to rule out early PHPTH would be the follow-up of our subjects in time. Further follow-up of our study population might therefore be necessary to give a clearer answer.

One main limitation of this study is the lack of anthropometric and social information (body mass index (BMI) and income level). This limits the interpretation of the results since obesity is known to increase PTH levels [10, 26]. In addition, dietary vitamin D and calcium intakes and the use of vitamin D supplements or of medications that can effect vitamin D metabolism were not recorded. However, the abovementioned factors seems to have little impact on the interpretation of the results since in a large cross-sectional Danish study, only 16% of the variability of PTH is explained by 25(OH)D, age, BMI, and daily calcium intake [8]. Finally, and because our study includes only subjects with an eGFR ≥60 ml/mn, no further ascertainment can be made regarding reference PTH values in patients with CKD.

## 5. Conclusion

A reference value for PTH was established for the first time in a sample of the Lebanese population using two different generation of PTH assays after excluding vitamin-D-deficient subjects. With both methods, the Lebanese subjects had higher PTH compared with the reference range provided by the manufacturer. We also confirmed that using a 25(OH)D cutoff of 20 ng/ml instead of 30 ng/ml did not change the reference range. Our study highlights the fact that, regardless of the assay used, each laboratory should establish its own normative PTH values in collaboration with clinicians in order to avoid under- or overestimation of PHPTH diagnosis. Since several studies have indicated that PTH levels in the upper limit of the normative reference level have a harmful effect on cardiovascular risk [27] and mortality [28], the clinical significance of the higher PTH levels in our population should be further investigated.

## Figures and Tables

**Table 1:** Baseline clinical and biological characteristics of the overall sample, men, and women.

	Overall sample *N* = 341	Men *N* = 110	Women *N* = 231	Test	*p* value
Age (years)	43.6 ± 11.8	45.7 12.2	42.5 ± 11.5	*T*	0.021
Creatinine (*μ*mol/L)	64.3 ± 13.1	76.2 ± 12.6	58.6 ± 9.0	*T*	<0.001
eGFR (ml/mn)	105.4 ± 13.7	102.5 ± 13.9	106.7 ± 13.4	*T*	0.008
Calcium (mmol/L)	2.40 ± 0.08	2.42 ± 0.07	2.40 ± 0.08	*T*	0.047
PTH 2^nd^ G (pg/mL)^*∗*^	48.9 [34.9–66.0]	46.5 [37.8–66]	51.2 [33.5–68.0]	*U*	0.788
PTH 3^rd^ G (pg/mL)^*∗*^	23.9 [17.6–30.5]	23.1 [18.6–30.6]	24.5 [17.2–30.4]	*U*	0.798
25(OH) D (ng/mL)^*∗*^	27.5 [23.9–32.6]	27.2 [24–32.6]	27.6 [23.8–32.8]	*U*	0.840

Data are expressed as mean ± SD or median and its interquartile range (Q1–Q3) PTH and 25(OH)D. ^*∗*^denotes variables with a significant departure of normality as detected by Kolmogorov–Smirnov and Shapiro–Wilk test and inspected graphically by quartile-quartile plots. *T* test: independent samples *T* test; *U* test: Mann–Whitney *U* test.

**Table 2:** Distributions of 2^nd^ generation PTH and 3^rd^ generation PTH according to dichotomized 25-OH vitamin D values (between 20 and 30 ng/ml and ≥30 ng/ml, respectively).

	2nd generation PTH (pg/mL)		3rd generation PTH (pg/mL)	
	20 < 25(OH)D < 30 (ng/mL)	25(OH)D ≥ 30 (ng/mL)	20 < 25(OH)D < 30 (ng/mL)	25(OH)D ≥ 30 (ng/mL)
Percentiles				
2.5	19.7	19.7	9.1	8.4
5	23.2	24.9	9.9	12.6
10	28.0	28.2	13.2	13.4
25	35.5	33.7	17.6	17.7
50	50.7	48.4	24.0	23.5
75	66.0	67.5	30.5	30.5
90	88.1	84.8	38.3	37.0
95	103.0	92.3	44.0	41.7
97.5	110.5	110.7	50.2	45.4
*p* value (*U* test)	0.696		0.917	

*U* test: Mann–Whitney *U* test.

## Data Availability

The data used to support the findings of this study are available from the corresponding author upon request.

## Abbreviations

PTH: Parathormone
25(OH)D: 25(hydroxyvitamin) D
BMI: Body mass index.
